# Extracts

**Published:** 1882-10

**Authors:** 


### Extracts.

---

American Gynecology, from an English Standpoint. —Simpson devised the slitting of the womb for sterility, but when transplanted into America, his operation, in the hands of Dr. Marion Sims, became one for the cure of most diseases of women. It is difficult for us simple-minded Englishmen to withstand American advocacy of surgical operations. They speak with the prestige of ovariotomy, successful after having been pooh-poohed for a quarter of a century by all the great surgeons of Europe. They dazzle us by prodigious statistics, of which cases given in detail are few, while failures find no place; so, when Dr. Marion Sims told us that in a short time, in one hospital, he had cured 500 women, of all sorts of uterine diseases, by slitting the cervix up to the body of the womb, the more experienced amongst us were very much astonished while the younger men rushed off to the instrument maker's, and followed the new light at the slower pace suitable to our placid nature. They soon found, however, that the operation did not fulfill Dr. Marion Sims' promises; that it was sometimes attended by very serious accidents; and then they heard that Dr. Sims' most promising pupil was busy sewing up the wounds he had helped his teacher to divide.

Some time ago we heard again from America, that Dr. Emmet had discovered a new operation for the speedy cure of the ordinary run of uterine cases. This new operation was again supported by hundreds of cases, not only of his own, but also of his pupils; cases without definite particulars; and without mention of failures, dangers, or of deaths. As some of our American friends express surprise that we have not adopted this new operation with the same enthusiasm as we did Dr. Marion Sinus', it is worth while, considering what we are now asked to do.

I gave it to be understood, that until Simpson made his mark, somewhat too large a part was given to inflammation for the explanation of uterine pathology. Since then the tendency has been to undervalue the importance of this most important element of general pathology and of daily practice.

Dr. Emmet, for instance, does not take the trouble to discuss what relates to inflammation in his voluminous work; he simply asserts that the womb cannot be acutely inflamed, except after parturition; in his large practice he seems never to have met with cases of acute internal metritis, than which I have never seen a disease so sharply accentuated or less amenable to treatment. As for chronic inflammation of either the substance or of the lining membrane of the womb, he says it is a sheer impossibility. Thanks are due to any man who will frankly say what he thinks on any important matter; but, when writing 855 pages on diseases of women, to dismiss in two pages the relations of inflammation to diseases of the womb, is neither scientific, nor scarcely respectful to what has so long stood as the keystone of uterine pathology; but let us see what Dr. Emmet puts in its place.

Subacute or chronic inflammation of the cervix, which so often shows itself by uterine ulceration, is for him a myth, a delusion, and nothing but the everted rim of a more or less lacerated cervix. He considers the womb to be so little up to its work, as to be always lacerated by parturition, and that what has hitherto been known as ulceration, is nothing but uterine ectropion. This theory, however, cannot possibly explain the same kind of uterine ulceration often met with in married women who are sterile, and sometimes in single women; so I may ask to be credited when I affirm that endocervicitis and ulceration are often met with in women who have borne children, without being caused by laceration.

Dr. Emmet asks us to treat these comparatively mild cases by extensive division of the neck of the womb, sufficient paring away of tissue, and by re-sewing of the divided cervix.

But this is not all, while admitting that nature often heals whatever laceration may have occurred, he maintains that it does so after a bungling fashion, by means of a very untrustworthy kind of a cicatricial tissue, which is to be cut away, says the Master, "if the cervix be large, or causes neuralgia;" to be cut away, wherever found, and under all circumstances, say the pupils, for fear of future mischief.

So now, in America, the womb is to have no peace; if its mouth be surrounded by a rim of ulceration, a delicate and serious operation is to be performed; and however sound the tissues may be, the same operation is recommended, should it be possible to detect any trace of a long-healed laceration. Of the many patients attended by me, during the last thirty years, I cannot remember a bad case of caco-plastic cervix with endo-cervicitis that I have not been able to cure by preliminary inter cervical incisions, deep enough to drain long congested tissues, followed by potassa fusa cum calce, and the subsequent dressing with tincture of iodine, so I do not see why the risk of a much more dangerous operation should be run, even in bad cases, except in a limited number that I have not time to specify. We are not, however, allowed to reserve trakeloraphy for long-standing cases of caco-plastic cervix with endo-cervicitis; we are actually asked to adopt this operation as the best means of curing the comparatively mild cases of endo cervicitis and ulceration, when the cervix seems otherwise healthy and not much larger than usual. These cases can be cured in two or three months by such applications as the acid nitrate of mercury, or nitrate of silver, coupled with proper medical treatment, and it would be preposterous indeed, to treat them by trakeloraphy. At all events, the question is now before the profession, and the ever increasing number of highly intelligent general practitioners, who become our judges on ceasing to be our pupils, know well how to discount the hobbies of metropolitan authorites, and the decision may be safely left to them.

I have seized this opportunity of entering another protest against the too frequent use of the knife in the treatment of uterine affections, but it must not be supposed that I consider what precedes to be necessary for the safe-guarding of the interesting objects of my solicitude in the present work. No, they have fortunately reached that most enviable time of life when the womb is well nigh safe from the onslaught of American surgery and the more vexatious, permanent irritation of pessaries that do not fulfill their object. The comparatively peaceful tenor of pathology at the change of life has reacted favorably upon the author. He knows that he will no longer be called upon to oppose the crochets of esteemed fellow-laborers, and that in a perfect spirit of peace, he may set himself to describe the closing scenes in the life of woman.—*Edward John Tilt, M.D., in preface to The Change of Life.*

---

Foreign Bodies in the Ear.—From an unprejudiced consideration of the character of the foreign body and of the anatomical relations of the meatus, in which it lies, the rule for most cases certainly is that no immediate danger threatens, even if the body remained for a long time; and this most valuable *a priori* reasoning in regard to foreign bodies in the ear can be fully supported from the literature of the subject. The worst to be feared is that the meatus will become stopped up, and the hearing consequently diminished, either from the body itself closing the meatus or from a gradual accumulation of cerumen about it.

In all simple, uncomplicated cases, and especially recent ones, the best course is not to touch the body itself, but, laying the child upon the affected side, with the head downwards, as, for instance, hanging over the edge of a sofa, to give the head slight blows from above downwards. Small, flat, unadhesive and unimpacted bodies can, in very many cases, by such means be again brought through the orifice of the meatus. Sometimes the bodies will be found the next morning in the bed, if that is examined. That many foreign bodies fall out of the ear of themselves is shown from the fact that the meatus of children brought to the physician "on account of a foreign body in the ear" is often found free, although no efforts at extraction have been made, or if made, have shown no visible results.

The next most useful course is syringing with warm water. Any cerumen which was narrowing the space and holding the body will thus be removed, and the water getting behind the body may wash it outward. At least, after such syringing, the side position and downward movements of the head may be crowned with better success. The only proper contra-indication for this syringing is when we are dealing with large parts of plants, which swell readily, and where we have to fear that a very slight increase in their size would bring them in contact with the walls of the meatus, and so impact them.

In cases in which small beetles, fleas, bugs, and similar animals get into the ear, and cause pain by their movements, the best course is to drop in warm oil, or to blow tobacco smoke into the ear, and then to syringe with warm water. By such means the insect either beats a retreat, or is rapidly killed; at least, it is brought out living or dead.

In many cases, a slight raising of the foreign body from the wall of the meatus will make a space through which water from the syringe can pass behind, and so force it out.

A flat, sufficiently long, and blunt lever, such as can be easily made of wood with the penknife, is adapted for such manipulations, or a delicate probe, without a bulb point, the point of which has been curved, or even bent at an angle, can also be used. If the child is intelligent and quiet enough, attempts may be made with such instruments thrust between the wall of the meatus and the foreign body to fix it from behind, and thrust it outwards either the entire distance or partially, and then to complete the removal by shaking the head or by syringing.

A very useful method in some cases is to pass a wire loop, like that upon a Wilde's polypus snare, along the side of the body, and fixing it in the loop, to draw it out. This method is particularly good, because the instrument does not injure the neighboring parts, and has the advantage that it can be passed through a small space. The attachment of an adhesive substance to the foreign body, and allowing it to dry, thus forming a handle by which the offending substance can be withdrawn, is a perfectly harmless method and frequently successful. In addition, children can be brought to submit to it readily.

As a *conditio sine qua non* to the use of every instrument, even the simplest, in the meatus, absolute immobility of the child must be assured, which can never be depended upon without anæsthesia, if any attempts at extraction have already been made; and again, the physician should be familiar with the minute anatomy of the ear, at least so far as to be aware that the obliquely placed drum-membrane lies nearest the orifice of the meatus in its upper posterior part; finally, the meatus should be thoroughly illuminated, so that the foreign body, with its surroundings, are distinctly visible. If any one of these precautions cannot be taken, or if the physician has had too little practice in examining the ear, the only proper and advisable thing is to abstain entirely from the use of instruments; otherwise the present unimportant condition of the patient may be changed into an unfavorable or even serious one, from the foreign body being forced farther inwards or from an injury to the meatus or drum-membrane. The less adapted the instrument is for the ear, or the more complicated the surgical apparatus used, the more likely will a *damnum permanens et irreparabile* be done to the little patient, from a neglect of some of the precautions which are necessary even in the simplest manipulation of this organ.—*Anton Von Troeltsch, M.D., in Diseases of the Ear in Children.*

---

Sewer Traps, and Disease Germs in Sewer Air.—In the following pages, then, I assert—

1st. That, according to the germ theory of disease in the form for which during the past fifteen years I have been an earnest advocate, diphtheria, typhoid fever, scarlet fever, and probably other contagious diseases, are connected with, if not solely due to, the development of spores or germs of vegetable organisms in the human body.

2d. That these germs propagate in sewers and float to us on the sewer air, penetrating into our dwellings through water closets, sinks, stationary wash-stands, etc.

3d. I have just discovered that the reason our various ingenious traps fail to protect us against these fatal sewer diseases is that sometimes a layer of micrococcus and mycelium creeps along the interior of the contrivance until it forms a new depot of development in the slimy vegetable lining extended into the *inner* or *house side* of the trap, from which, without obstruction, its deadly germs may be given off into the very bed-chambers of its victims; therefore,

4th. The true method of obviating this danger is by sterilizing with slow currents or drippings of solutions of sulphate of iron, corrosive sublimate, arsenic, carbolic acid, etc., the whole interior of our waste pipes, just as the shores of the Dead Sea and the banks of certain small streams are sterilized by mineral ingredients, or poisonous metallic substances from manufacturing refuse, with which their waters are mingled.

As a remedy for offensive sewer emanations, solution of sulphate of iron (common copperas) has long been known, and I am strongly inclined to hope that in it, when *properly and systematically applied*, we have an agent capable of making the surface of waste pipes unfit for vegetable growth or life, and so rendering these conduits completely sterile, just as certain weeds may be killed and conquered by watering the ground where they grow with a strong saline solution.

Varied and prolonged courses of experimental inquiry will doubtless be requisite for deciding numerous minor details, and especially just how often waste-water pipes must be bathed with solutions of mineral substances, in order to secure the desiderata above indicated. But I am confident that the key to this momentous problem, of how to avoid infection from "sewer gas," or, more correctly, sewer air, entering our dwellings, is to be found in the principle of *so sterilizing* the whole interior of all pipes communicating with sewers, and, if possible, of the sewers themselves (by frequently irrigating them with fluids containing metallic compounds *poisonous* to plant life), that no vegetable organisms can propagate within them. Under such circumstances, *disease germs*, even if they penetrate for short distances through imperfect traps, will be powerless for evil, because a noxious element being introduced into their environment, the harmoniously favorable conditions necessary for their development and reproduction are disturbed.—*Joseph G. Richardson, M.D., in Philadelphia Med. News.*

---

The Subcutaneous Injection of Ether.—It should be more generally known that ether injected subcutaneously has a powerful stimulant effect, and is remarkably efficacious in cases of extreme depression of the powers of life. It has long been used to a limited extent in such cases, but increasing experience has enlarged the domain of its application. In adynamic pneumonia, in fevers when failure of the vital powers is threatened, in the puerperal state, in cases of thrombosis of important vessels, the injection of ether has been lately used with singular benefit. Besides, as a stimulant in conditions of depression, it has important applications as a hypnotic and local anodyne. In cerebral excitement and wakefulness, accompanied by depression of the arterial circulation, it is most useful. In the more chronic cases of superficial neuralgia, as sciatica, lumbago, intercostal pain, zoster, etc., ether injected in the neighborhood of the affected nerves often gives surprising relief.

There are contra indications to its use. It is not proper in the cases of cardiac depression due to chloroform or ether narcosis, and yet it has, in the confusion incident to such an event, been freely injected on the cessation of the cardiac or respiratory movements. Under similar circumstances, alcohol has also been freely injected subcutaneously, but this practice is equally improper—and both for the obvious reasons that these are synergistic agents. Ether, subcutaneously, is also not a suitable remedy when there is arterial excitement with power.

The technical details are simple. Ether must be injected with a glass or metallic syringe. Rubber and celluloid are damaged by it. As ether dissolves the oil with which the piston is lubricated, the syringe should always be put in order after ether has been injected. It is a useful precaution, also, to see that no particles of dirt or of leather are taken up with the fat. Vaseline appears to be the safest lubricant under these circumstances. From ten to sixty minims is the dose—fifteen minims being the quantity most frequently injected. Some smarting attends the operation, but if the operator is careful in withdrawing the needle to press on the orifice tightly, to prevent the ether escaping, much smarting will be thus obviated. A puffy swelling is caused by the vaporization of the ether, but this presently subsides, and only rarely is an indurated knot formed. An anæsthetic and analgesic area of limited extent surrounds the puncture.

The ether used should be of good quality—as good, indeed, as that now employed for inhalation. The number of times injected will depend on the character of the case, but there appears to be no reason why it may not be injected frequently. Three or four times a day has been the rate in cases of adynamic pneumonia. When sudden, extreme depression of the heart is to be overcome, ten or twenty minims can be injected every five minutes, until some result is reached.

The systemic effect is that of a stimulant; the action of the heart is increased, the surface grows warm, and the nerve centres and the organs of the body in general functionate more quickly and powerfully. The curative results of the subcutaneous use of ether are not only different in degree, but in kind, from the stomachal administration of the same agent. This fact must be recognized to obtain a correct notion of the utility of this practice.—*Philadelphia Med. News.*

---

TREATMENT OF FRACTURE OF THE SKULL WITH DEPRESSION.—On this important and interesting subject the *Lancet* makes the following remarks:

It is not improbable that in the operative treatment of injuries and diseases of the skull, some of the most important advances in surgery in the immediate future will be made. We now operate freely on deformed bones and diseased joints; operations on the abdominal viscera are now undertaken, which but a few years ago were looked upon as altogether beyond the bounds of the practicable; and the therapeutic advances that have made these procedures so successful may be equally well applied to the surgery of the head But accuracy in diagnosis is an essential condition of successful treatment, and as the recent discoveries of the special function of individual portions of the surface of the brain are rendering exact diagnosis of superficial brain lesions possible, it is not rash to foretell that surgery will keep pace with the science of diagnosis. The surgical treatment of depressed fracture of the skull has been formulated into the proposition that when accompanied with evidence of compression of the brain, an operation to remove the depressed bone should be undertaken at once. This is the general if not the universal practice among British surgeons. But latterly, some inclination to extend the use of the trephine has shown itself in more than one quarter. The reasons for this extension are that the operation, when properly conducted, and especially when carried out with due precaution against septic infection of the wound, is not a very dangerous one, and if we may argue from Dr. Yeo's experiments on monkeys to man, we may say that the use of the trephine adds very little, if at all, to the gravity of a case. Again, surgeons from time to time see cases where the results of a post-mortem examination show that a life might have been saved by the timely use of the trephine. In Paris the doctrine was first promulgated that the trephine ought to be used in *all* cases of *compound* fracture of the vault of the skull, whether actual depressions of bone, or symptoms of compression, be present or not. And lately in America, it has been urged by Dr. Gunn that in *all* cases of depressed fracture, with or without symptoms of compression, and whether simple or compound, the depressed bone should be elevated. While we fully believe that the operation of trephining has fallen into undeserved disrepute, and might with advantage be resorted to more freely, we think that Dr. Gunn's proposal is unsound and dangerous. It is a familiar fact to any surgeon of large experience that patients who have received depressed fractures go through life quite unaffected by them, and while such is the case no appeal to cases in which the course of events is different ought to induce a surgeon to submit all cases alike to operation. For there is not conclusive evidence before us that the evil effects of depressed fractures, which often arise, may, without giving rise to symptoms, advance to such a degree as to render trephining useless. There appears, then, to be no sufficient ground for formulating the rule of practice otherwise than as at present, but it should be more liberally interpreted, close watch for symptoms of local mischief shou'd be kept up, and the trephine resorted to as soon as any such symptoms declared themselves, even though slight, and with strong hopes of a successful issue, instead of, as at present looking upon it as a *dernier ressort*, and using it timidly and with gloomy foreboding.—*Med. and Surg. Reporter.*

---

HEATON'S OPERATION.—Now the next case is one which you have seen before, and it is a man on whom I propose to operate again by Heaton's method for the radical cure of hernia. I have already done this operation twice upon him, with the result of only a very imperfect obliteration of the inguinal canal. He is one of the three or four cases in which I have tried this method, and in none of these cases has the result been entirely satisfactory. But whenever a surgeon of high standing proposes some new operation with which he has himself had good success, I think that we should always give these new methods a fair trial, and that we should perform the operation in precisely the same way in which he has done it. So we feel that this method of Heaton's has sufficient testimony in its favor to make it worth our while to try it thoroughly. The operation consists in injecting a solution of white oak into the areolar tissue of the inguinal canal, with the idea that a sufficient amount of inflammation will be set up in this way to effect a permanent closure of the canal. The solution injected consists of the fluid extract of white oak, with a certain amount of alcohol and ether, to which is added a little morphia. But this addition of morphia I do not like, because if this drug is present in any considerable quantity it bars you from using the solution to any extent you wish. Suppose, for instance, that twenty drops of the solution contain the usual quantity of morphia for a single dose, then if I wish to deposit three times twenty drops I am prevented, because I fear giving an overdose of the anodyne. And besides, if the morphia is needed, it can easily be injected into the arm in the proper dose.

For injecting the solution I use an instrument which varies somewhat from the usual form, and which I suggested and had made for myself. The idea in it is to have a hollow needle with a blunt point of steel which is nevertheless somewhat acumenated, so that it can be run about in the loose areolar tissue of the canal as far and as freely as a sharp needle, while the danger of wounding the adjacent parts is avoided. With a little care you can determine exactly where the point of the needle is going, and you can easily avoid wounding the vein, which is the chief point of danger here.

This man has been operated on three times before this, and two of them were done by me; and there is now a certain amount of solid material effused here, and there is some degree of obliteration of the canal. But I think it needs to be further obliterated by the injection of more of the solution.

*Operation.*—My syringe now contains twenty minims of the fluid, and I will be contented with this amount for to-day. At the point where I intend to insert the needle I first make a small cut through the skin with a sharp knife, and then through this opening I push the needle into the canal, directing it upwards and outwards, and then moving it in any direction I like. And by taking this precaution I can feel the utmost certainty that I have the needle in the canal. Now I deposit a few drops at three different points in the canal: one near the external ring anteriorly, and another directly opposite to this and posteriorly, and another near the internal ring and anteriorly, as nearly as I can locate them. An ordinary broad bandage will be all the dressing needed at present.—*T. M. Markoe, M.D, in Med. Gazette.*

Examination of the Naso-Pharynx in Children.—The inspection of the naso-pharynx by rhinoscopy is only possible in exceptional cases in children under ten years of age, but a tactile examination with the finger is readily made, even by those whose fingers are short. The head of the patient should be supported from behind with one hand, while the forefinger of the other hand is passed backwards into the open mouth; if the palate is drawn up, and the entrance to the naso-pharynx thus closed, wait quietly, avoiding any pressure with the finger. As soon as the patient takes a deep breath, the velum palatinum will sink, and it is then possible to pass the finger upwards, when, with careful movements, it is possible to feel of all parts of the naso-pharyngeal cavity, on the sides, forwards, backwards and upwards, and in some cases it is even possible to pass the finger through the choanæ into the nasal cavity. With some practice it is possible quickly and with the finger alone, to gain a sufficiently good idea of the size of those cavities, of the thickness and condition of the mucosa, upon which, as we have already shown, there are often extensive infiltrations, or partial hypertrophies, which can be felt as ragged or knobbed prominences.

When such exist upon the vault of the pharynx, they usually arise from the third tonsil, tonsilla pharyngea, which is situated at this spot, and, in childhood, is very large and always provided with fissures and irregularities. The finger on being withdrawn is very often bloody, even after a quiet and undisturbed examination, a proof of the succulence and congestion of the tissues; if soft granulations are present in any numbers, nose bleeding is often produced. The more quietly the patient breathes during this palpitation, the more easily and thoroughly it can be made. It is rarely painful, and intelligent children usually allow the later examinations without opposition, although the first may be opposed.

Care should be taken, not only to fix the head firmly, but the hands should also be held fast, which can be most easily, done by having the child sit in the lap of an adult, who can embrace it with both arms. If the patient does not willingly open his mouth, the nose should be tightly closed. The teeth of the patient are much less likely to injure the fingers of the examiner than would be supposed, but it is sometimes advisable to protect the first phalanx with a bit of rubber tubing, and it is desirable also to take a position beyond the reach of the patient's feet, the reflex movements of which sometimes produce disagreeable blows —*Anton Von Troeltsch, M.D., in Diseases of the Ear in Children.*

---

THE SMOKE TEST FOR TENEMENTS.—Under the head of "Nuisances," we had in dealing with structural defects often to contend with defectively jointed soil pipes and drains in connection with sinks, wash-hand basins, and water closets, which exposed the dwellings where they were placed to the invasion of sewer-gas, and these being in many cases inaccessible from the stupidly persistent practice of laying them beyond convenient reach, baffled the efforts of the most skillful in applying a proper remedy.

But we have now to a very considerable extent overcome this difficulty by the free use of the smoke test—a simple and most valuable invention for the discovery of dangerous leakages.

This test is applied by a small machine with powerful fanners, which blow the smoke of ignited cotton waste, into the whole drainage system of the tenement operated upon, by openings in the soil pipes, or otherwise, and which in due time issues at all imperfect joints and connections, disclosing their position with perfect accuracy. The operation is most successful when it is practicable to expose all the pipes along their course from the house to the main sewer, and in all cases every effort is used to accomplish this.

The inspector of the district in which the drains are situated, superintends the process in presence of a tradesman employed by the owner, who notes the leakages, and thereafter closes them up, when another test is applied and the absence of smoke-issues along the line proves the completion of a satisfactory remedy. A written report is then given to the owner, and is greatly appreciated by both owners and occupants.—*Glasgow Sanitary Inspectors' Report.—Sanitary Engineer.*

---

Treatment of Vomiting in Pregnancy.—Prof. C. Braun, of Vienna, reports a case to which he was summoned, the patient being regarded as moribund. She was in the first half of her pregnancy and extremely reduced in consequence of intractable vomiting. The physician in charge had decided on producing premature delivery as a last resource. Braun, who has often opposed this practice, decided instead to bathe the vaginal portion of the cervix with a ten per cent. solution of nitrate of silver. This was done, and the surface quickly dried to prevent further cauterization. The success of the treatment was so immediate and so great that an hour afterward the patient enjoyed a meal of roast veal, and there has been no vomiting since. Prof. Braun thinks that hyperemesis should be expunged from the list of indications for artificial abortion. He has never seen a case of death from hyperemesis. In France, where abortion is frequently induced to relieve this symptom, it is found that the vomiting is thereby stayed in only forty per cent. of the cases, while in ten per cent the operation has been fatal. *Allgem. Wiener Med. Zeitung.—American Practitioner.*

---

Obstetrical Forceps.—It may not be out of place here to remark that I am no advocate for the indiscriminate and frequent employment of forceps in obstetrics, and venture to observe, from what I have seen in hospital and city and country practice for many years, that more permanent injuries have been inflicted upon the mother than benefits have resulted from their use, especially in the last decade, and language is inadequate to express my repugnance to, and cendemnation of, their present reckless abuse. First employed by a *quack* and the secret retained for years, he is not the only one who too frequently—and too often, it is feared, for effect, or, as sometimes avowed, "to save time"—resorts to them. That conditions arise where their employment becomes not only imperative but indispensable to preserve the life of the mother and child, or to abbreviate protracted suffering, is a foregone conclusion, and were their use reserved for such we should hear less of their abuse, and the pernicious consequences, either immediately or remotely, thereby entailed. The trite but true adage is always applicable—"a meddlesome midwifery is bad."—*Jas. L. Tyson, M.D., in Phila. Med. Times.*

---

Politics and Medicine.—As a profession, doctors think it undignified to have much to do with politics. As a whole, they are not as big tax-payers as they ought to be, but numerically strong, intelligent and influential. Unfortunately, other of the better elements of society hold themselves aloof from mingling in what they term the filthy pool of politics. This condition ought not to be, if we want to break down bossism and machine politics, to defeat vicious and secure good legislation. 'Twould be a misfortune for all doctors to think alike, but if each will follow his convictions into the ranks of that political party nearest his liking, then exert his influence toward sending intelligent, representative men to the legislature, we may secure the enactment of such laws as will protect the people, and convince them that we have been impelled by motives other than a desire to attain selfish ends.—*Dr. Crumpton, in Pacific Med. and Surg. Journal.—Chicago. Med. Jour. and Ex.*

---

Medical Morality.—The Alumni Association of the Albany Medical College has published the Proceedings of their Ninth Annual Meeting. President Vanderveer delivered the address, and made an earnest plea for the *moral* improvement of the profession. He most justly says:

"Medical colleges cannot do all that is needed at the present time to improve the condition and standing of what we so love to mention as one of the three learned professions. They are making some effort in various directions, but the facilities and inducements in some portions of the country to organize new schools are such as to cripple seriously the good that is being done. I am sure that all the quackery exercised in the present day does not do as much harm to the practice of medicine as do the self-advertising, immoral and unworthy men in our ranks." — *Philadelphia Med. and Surgical Reporter.*

---

DARWIN.—In the current number of the *Westminster Review* there is contained a careful and critical essay on the late Charles Darwin and his works. We may be forgiven for reproducing the following admirable judgment on Darwin:

"The life of Darwin is an epic. The object of his highest aspirations—of his earnest and diligent pursuit—was Truth. He followed ever where she led, or seemed to lead, and even when the path diverged from the beaten track and became entangled with briars and thorns, he still pushed on, undeterred by perils, seeing the beacon light afar. Luxury and ease invited him, but he rejected their allurements, and still pressed on. Enemies opposed his progress, and assailed him with every weapon in their power; but, like a skillful general, he allowed them to spend their strength in vain upon a fortress which he knew to be impregnable, and he triumphed. Yet, even in the hour of triumph, he never lost that modesty which is the finest attribute of a seeker after truth, and even his bitterest enemy could never accuse him of arrogance or discourtesy."—*Medical Press and Circular.*

---

OBJECTIONABLE ANÆSTHESIA. — Western women are sharp; but the Plattsmouth (Neb.) female is entitled to the premium for smartness. The other day she went into a shoe store to buy a pair of shoes. The clerk was in the act of sprinkling some chalk-powder inside, so they might slip on easily. She glanced furtively at him and remarked: "I know what you are doing." The genial clerk smiled acquiescence. She slid toward the door, and said, in tones that startled his nerves: "You can't chloroform me, mister; I was fooled once before, and I'm blamed if I'll be again." And she left without the shoes.—*Cincinnati Lancet and Clinic.*

---

ACTION OF ALKALIES ON PEPSIN.—The *Druggists' Circular* mentions that it is said that alkaline solutions of pepsin lose their digestive power; but whether they recover it or not when again acidified, has not been proved. Opinions appear to differ.—*Therapeutic Gazette.*

